# Bilateral De Quervain Syndrome after Aromatase Inhibitor Administration: A Case Report and Review of the Literature

**DOI:** 10.1155/2012/810428

**Published:** 2012-04-11

**Authors:** Konstantinos Papadimitriou, Panteleimon Kountourakis, Emmanouil Morakis, Vassilios Vassiliou, Vasileios Barbounis, Alexandros Ardavanis

**Affiliations:** ^1^Department of Medical Oncology, BOC Oncology Centre, Nicosia, 2006 Strovolos, Cyprus; ^2^First Department of Medical Oncology, Saint Savvas Anticancer Hospital, Athens, Greece; ^3^First Orthopedic Surgery Department, Evangelismos Hospital, Athens, Greece; ^4^Department of Radiation Oncology, BOC Oncology Centre, Nicosia, 2006 Strovolos, Cyprus; ^5^Medical Oncology Unit, Hipokrateion Hospital, Athens, Greece

## Abstract

Aromatase inhibitors are widely used as one of the main treatment options of both early and advanced hormone receptor-positive breast cancer in postmenopausal women. Unfortunately, musculoskeletal symptoms are often presented in patients treated with aromatase inhibitors (AIs), and, although the pathogenesis is unknown, postulated mechanisms have been described. Herein, to our knowledge, we present the first report of bilateral De Quervain syndrome related with AIs therapy with a review of the relevant literature.

## 1. Introduction

Recently, aromatase inhibitors (AIs) have substituted tamoxifen as the preferred adjuvant treatment of both early and advanced hormone-responsive breast cancer in postmenopausal women. AIs have been proven to be more effective than tamoxifen, showing improved outcome and lower incidence of distant recurrences. In addition, they have a more favorable toxicity profile compared to tamoxifen, with lower rates of thromboembolic events and endometrium cancer occurrence. However, their main side effects include bone mineral density reduction and musculoskeletal pain symptoms. These musculoskeletal symptoms present either as specific conditions as carpal tunnel syndrome (CTS), tenosynovitis, tendinopathies, and polyarthralgias of the hands and fingers or as nonspecific mono- or oligoarthralgias. The incidence of these symptoms has been reported to vary between 5 to 35%, and almost one-third of these patients discontinued therapy due to severity of the symptoms.

## 2. Case Report

 A 48-year-old postmenopausal woman with an early breast cancer (T1b, N0, M0, ER(+), PgR(+), HER-2(−)) was referred to our department in December 2009. She was initially treated by lumpectomy and subsequently by oral letrozole. Nine weeks after treatment's initiation the patient claimed progressive worsening of bilateral pain to the radial surface of the wrists and important functional impairment. Finally, pain caused rising from her sleep.

On clinical examination, severe sensory paraesthesias presented to the dorsal surface of the wrists and to the base of thumbs. Inflammatory signs became apparent as well as palpable nodule with a “bounce” feeling at the movement of the thumb. Moreover, the clinical evaluation revealed bilateral positive Finkelstein's test, which is characteristic of De Quervain syndrome (stenosing tenosynovitis). This test in combination with the described anatomic location of the pain on the thumb side of the forearm, about two centimeters below the wrist, rules out the diagnosis of intersection syndrome. Furthermore, the negative imaging studies performed (X rays) excluded the diagnosis of osteoarthritis of the first carpometacarpal joint. In addition, a negative electromyogram excluded the diagnosis of Wartenberg's syndrome. Additional laboratory studies were performed and exclude systematic inflammatory process or findings indicative of autoimmune disease. The patient reported no use of other medications or other health conditions. Finally, it was confirmed that the patient did not have overuse injuries due to daily repeated movements of the thumb, which could cause stress tendinitis from lassitude.

 Initially, the patient was treated with compression bandages to immobilize the wrists and oral nonsteroidal anti-inflammatory drug, which failed to relieve the symptoms. Consequently, interruption of letrozole was recommended and, five weeks later, the patient reported progressive improvement up to complete regression of symptoms. After two months of interruption of letrozole and total disappearance of symptoms, the patient was started on exemestane and the syndrome did not reappear after start of the latter. To our knowledge, this is the first report of bilateral De Quervain syndrome probably related with AIs therapy.

## 3. Discussion

Musculoskeletal symptoms are often described in patients treated with AIs. Although the term “arthralgias” is commonly used, the lack of uniform assessment of these symptoms in different studies created the large diversity in the clinical entities described. AI-related arthralgia has been reported throughout the whole musculoskeletal system but is particularly common in the anatomic region of wrist joint [1–3]. Relevant clinical conditions reported include CTS, tenosynovitis, “trigger finger,” morning stiffness, as well as tendinopathies, polyarthralgias, or nonspecific mono- or oligoarthralgias and effusions of the hand and wrist [4–7]. Daily activity restrain, related to musculoskeletal disturbances from AIs use, has not been studied systematically, and it is often underestimated. In some cases, it has been the reason for discontinuation of treatment and constitutes serious cause of kinetic loss [1]. In a small group of patients, switching to another AI allows continuation of therapy and therefore maintains the expected benefit in disease outcome.

The incidence of musculoskeletal events varies between 5% and 35%, with CTS and “trigger finger” being the most frequent [8]. In addition, estrogen deprivation caused by AIs induces bone mineral density decrease and fragility fractures which may represent another common cause of musculoskeletal symptoms.

 Unfortunately, the heterogeneity of symptoms is proportional to high diversity in imaging, laboratory, and pathological findings, rending diagnosis and understanding of AI-related joint pain pathophysiology a difficult task. In fact, some studies involving ultrasound or magnetic resonance imaging (MRI) have documented tendon, tendon sheath, and synovial changes observed in patients with severe AI-induced musculoskeletal symptoms. On the other hand, a large body of clinical trials in patients treated with AIs relies the diagnosis of relative situations like trigger finger, intersection syndrome, or “arthritis” on clinical findings without essential report of imaging results [3, 4].

 More studies confirmed significant thickening of the digital flexor tendon synovial sheath on MRI [5, 6]. Furthermore, no abnormalities have been usually seen with radiographs or bone scintigraphy [5]. Though, it is to be noted that the results for arthritis findings in imaging studies may vary depending on underline situations such as the degree of osteoporosis, osteoarthritis, or rheumatopathies [5, 6]. Nerve conduction studies and EMG testing, in patients with AI-induced symptoms suggestive of CTS, have been also performed; varying levels of median mononeuropathy at the wrist have been reported. Finally, studies evaluating systemic inflammatory and autoimmune markers in patients with aromatase inhibitor-associated tendinopathy, such as ESR, CRP, RF, antinuclear antibody, anti-double-stranded DNA antibody, and anticyclic citrullinated peptide antibody, have not identify a correlation [5, 6]. Only one prospective study focusing on this aspect showed a minor elevation of systemic inflammatory markers [7].

 Although the pathogenesis is unknown, postulated mechanisms have been described. A rational explanation for these symptoms could be related to the fact that estrogen levels interfere with body hydratation status. It could be speculated that they keep the body “moist” and that, without estrogen, everything dries up, including the joints. Without “lubrication,” the joints get irritated and then swollen. Unfortunately, pathogenesis of this condition is much more complicated and not well known. Recently, there is a tendency of classification of these symptoms into two general categories: (a) joint pain as in arthritis and (b) a more generalized discomfort involving muscle and skeleton, what the bibliography calls aromatase inhibitor arthralgia (AIA). Extensive research is evolving for its understanding, and different approaches are reported.

 AIA is more likely related to estrogen deprivation that leads to suspension of their antinociceptive effects. Mechanisms of joint pain include enhanced nociception due to estrogen depletion. Evidence exists that estrogen may have an antinociceptive and pain-modulating effects, and it is known to have effects on opioid-related receptors in the central nervous system [8]. Indeed, in vitro data confirm that estrogen-receptor-positive cells express preproenkephalin messenger RNA, and also estrogen given to ovariectomized animals increases enkephalin transcription in the spinal cord [9]. These neurons provide critical inhibition of nociceptive neurons.

 Another study proved that opioid-receptor-like 1 (ORL_1_) mRNA is present in the majority of estrogen receptor mRNA-containing neurons. The study concludes that sex-related differences in the ORL_1_-receptor gene expression in the trigeminal nucleus caudalis appear to be affected by estrogen levels [10]. Estrogen receptors are also present in brain as estrogen treatment stimulates the release of endogenous opioids that activate mu-opioid receptor (mu-OR) in the limbic system and hypothalamus providing a “neurochemical signature” of steroid activation of these circuits [11]. Taken together, these in vitro data imply an even more direct effect of estrogen on opioid generation locally. These observations are further supported by clinical trials for nociception variations in women during their menstrual cycle and menopause.

 In fact, joint symptoms in postmenopausal women described as “Menopausal arthritis” were first described in 1925 [12]. Women during pregnancy have elevated thresholds for painful stimuli in the presence of increased levels of estrogen [13]. Furthermore, in nonpregnant animals in which pregnancy levels of estradiol and progesterone are simulated, pain thresholds rise [14]. In women treated with hormone replacement therapy, a significantly lower chance for developing musculoskeletal symptoms was reported [15]. These data support the hypothesis that high levels of gonadal hormones suppress pain sensitivity. However, hormone replacement therapy has not proven its efficacy as adequate treatment for arthralgia in postmenopausal women [16].

 There is no doubt that the specific effects of aromatase-containing cells on nociceptive fibers are less understood than the direct effects of estrogen. In breast cancer patients under treatment with AIs, joint pain appears also to relate with the “anti-nociceptive effects” of estrogens and consecutive increase of pain threshold [14, 17]. Opposite though were the results of an early meta-analysis in healthy women across their menstrual cycle, which demonstrate much better pain tolerance during times of lowest estradiol and progesterone levels of the menstrual cycle [18]. Differences in the methodology of pain registration in the literature may explain some of the conflicting results.

 No specific effects of estrogen on articular structures have been reported, but it influences inflammation and neural processing of nociceptive input [14]. There are also data available for inflammatory process activation via autoimmune pathways, after therapy with AIs [19, 20]. However, as previously noted, updated data in patients on AI treatment do not confirm an increased incidence of autoimmunity or raised systemic inflammatory markers. Little data exist on inflammatory effects of estrogens within the joint, but tissue-specific effects of estrogen on inflammatory cytokines have been reported [14, 21]. Characteristically, while estrogen enhances nitric oxide production in endothelial cells, it inhibits nitric oxide and prostaglandin E2 production in microglia [22]. There is also evidence that the proinflammatory cytokines, Il-1, Il-6, and TNF-alpha, are elevated in the first few years after the menopause, when estrogen levels are lower and the incidence of joint symptoms is high [23].

 When a joint is inflamed, mediators such as prostaglandins and bradykinin could also activate receptors on peripheral nociceptors, enhancing their sensitivity to pain and also neuron's nociceptive receptor fields enlarge [23]. Probably inflammation in the joint could account for enhanced nociception that occurs with estrogen depletion.

 In a study on synoviocytes, in which aromatase was shown to convert androstenedione to oestradiol, Il-6 production was reduced [24, 25]. Therefore, reduction of oestradiol may promote local inflammatory changes in the joint by this mechanism. Evidence exists that the proinflammatory cytokines, Il-1, Il-6, and TNF-alpha, are spontaneously elevated in the first few years after the menopause a time when the natural incidence of joint symptoms is high [26]. Indeed, it has been suggested that time since menopause may be an important predictive factor for AIA, and this may be linked to cytokine activity [26, 27].

 A different approach on AIA pathogenesis is that of a recent study, correlating the role of estrogen depletion on melatonin secretion and joint pain. The authors make two interesting clue points: the observation that joint pain is associated with virtually all estrogen-depleting breast cancer treatments suggests that the cause is broader than the case of AIs, and, second, usually the discomfort is worse in the morning and the strongly circadian nature of these symptoms suggests circadian hormone involvement. Estrogen depletion could increase pineal melatonin, and, in addition, the ability of light to suppress pineal melatonin is more variable than once thought and that an altered melatonin cycle is associated with rheumatoid arthritis patients, where identical circadian symptoms present. It is hypothesized that when AIs decrease estrogen levels, light-induced melatonin suppression loses efficacy, leading to an abnormal melatonin cycle as seen in rheumatoid arthritis patients, producing the symptoms of morning stiffness [28]. This study suggests that suppressing melatonin production by light exposure will reduce the pain in women on AIs. Interestingly, in one patient, symptoms were relieved by spending lots of time outside.

 One more field of clinical research for AIA pathogenesis, independently of the possible mechanism, is the possible contribution of an extensive spectrum of diseases that typically induce musculoskeletal symptoms. There is a high background level of joint symptoms in the peri- and postmenopausal female population and it is important to consider this when evaluating the incidence and etiology of AIA [29]. It is known that the incidence of joint pain peaks at 50–59 years in the general population. Some preclinical studies have shown a protective effect of estrogen in arthritis and on proinflammatory genes. Clearly, there are several possible causes of arthralgia in a nonbreast cancer population, which can make it difficult to elucidate one particular cause. Other studies have also shown that BMI is associated with an increasing risk of joint pain. The incidence of pain in at least one joint has been as high as 49% [30].

 The increased prevalence of carpal tunnel syndrome is one of the clinical expressions of AIA. A possible explanation could be compression neuropathy of the median nerve due to the presence of fluid around the flexor tendons and edema of the wrist, as previously noted. In a relative study of nonbreast cancer patients undergoing surgery for carpal tunnel syndrome, tissue from the transverse carpal ligament and synovium was examined and found to highly express ER and PR. This observation potentially reveals a role of these receptors in the pathogenesis of carpal tunnel syndrome. Interestingly, the number of ER-positive cells increased with age to a peak at 55–70, decreasing thereafter [31].

 To our knowledge, this is the first report of bilateral De Quervain syndrome related with an AI therapy. Switch to another AI due to severe side effects could be an option. Further research will probably give prospects for new predictive markers, while the study of pathological changes in concrete anatomic sites of tendon sheath will probably lead to comprehension of parallel mechanisms of AIs action and tailored made symptomatic therapy.
